# Multi‐contrast X‐ray microtomography of human lung specimens with an extended field‐of‐view

**DOI:** 10.1002/mp.70335

**Published:** 2026-02-19

**Authors:** Harry Allan, Adam Doherty, Carlos Navarrete‐León, Oriol Roche i Morgó, Yunpeng Jia, Charlotte Percival, Zoe Hagel, Kate E. J Otter, Chuen Ryan Khaw, Kate HC Gowers, Helen Hall, Sam M. Janes, Fleur Monk, David Moore, Ryoko Egashira, Joseph Jacob, Marco Endrizzi

**Affiliations:** ^1^ Department of Medical Physics and Biomedical Engineering University College London London UK; ^2^ X‐ray Microscopy and Tomography Laboratory The Francis Crick Institute London UK; ^3^ UCL Respiratory University College London London UK; ^4^ Department of Cellular Pathology University College London Hospitals NHS Foundation Trust London UK; ^5^ CRUK Lung Cancer Centre of Excellence, UCL Cancer Institute University College London London UK; ^6^ Hawkes Institute University College London London UK; ^7^ Present address: Now with the Diamond Light Source Harwell Science and Innovation Campus Didcot OX11 0DE UK; ^8^ Present address: Now with the Department of Life Sciences Birmingham City University Birmingham B4 7BD UK; ^9^ Present address: Now with the Department of Respiratory Medicine King's College Hospital London SE5 9RS UK

**Keywords:** multi‐scale imaging, virtual histology, X‐ray phase‐contrast

## Abstract

**Background:**

Phase‐based X‐ray microtomography is a powerful technique capable of quantitative volumetric imaging of lung tissue in health and disease. The maximum sample size is however limited by the fixed sizes of detectors and optical elements. Thus while high‐resolution imaging can offer valuable microscale insights, it can be difficult to interpret without the context of the surrounding tissue. We propose a multi‐contrast and multi‐scale approach, combined with an offset geometry to extend the field‐of‐view (FOV).

**Purpose:**

FOV limitations make it a challenge to simultaneously achieve high spatial‐resolution and image large samples. Our method doubles the possible FOV achievable for a given spatial‐resolution, in a way compatible with multiple scales and imaging systems.

**Methods:**

Multi‐contrast whole sample volumetric images are acquired using a beam‐tracking X‐ray phase‐contrast imaging(XPCI) system. Following this, a section of the same sample is imaged at higher resolution using an X‐ray microscope with propagation‐based imaging. The FOV of both methods is doubled using an offset center‐of‐rotation geometry, followed by weighted analytical reconstruction.

**Results:**

We present exemplary multi‐contrast reconstructions of resected human lung tissue at 10.5 μm voxel size across a 4.3 cm horizontal FOV, and at 450 nm voxel size for a 2.7 mm section of the same sample. This enables the visualization of a range of features, from the macro to the cellular scale.

**Conclusions:**

We demonstrate a versatile method to image large samples without sacrificing spatial‐resolution. This method is directly compatible with complementary implementations of XPCI, and is easily adapted to a range of other systems.

## 1 INTRODUCTION

X‐ray computed tomography (XCT), has proven to be an indispensable tool for the non‐destructive 3D inspection of samples in a range of fields including medical sciences, materials engineering, and metrology.^1^ In the study of lung disease, micro computed tomography (micro‐CT) has allowed detailed virtual histology of morphological and structural changes due to fibrosis,^2^ Covid‐19,^3^ and adenocarcinomas.^4^ Access to volumetric information enables 3D quantification of disease biomarkers,^5^ which can be demonstrably more sensitive than their 2D counterparts,^6^ enhancing the understanding of disease and the effects of treatment.

Despite its widespread adoption, the application of micro‐CT to low‐density materials, such as lung tissue, still suffers from their inherently low X‐ray attenuation cross section. X‐ray phase‐contrast imaging (XPCI)^7^ is capable of improved contrast‐to‐noise ratio (CNR) compared to conventional attenuation‐based imaging, and has thus been proposed as a solution to this problem. While typically requiring X‐ray beams with high levels of spatial and temporal coherence,^8^, ^9^ several techniques have been developed to enable the use of XPCI techniques with conventional sources, increasing their availability in laboratory and clinical settings. A number of these methods utilize optical elements in the beam,^10^, ^11^, ^12^, ^13^, ^14^ which structure the illumination in a way that allows the retrieval of multiple contrast‐channels, namely attenuation, differential phase (refraction), and dark‐field. The size of this optical element, alongside the size of the X‐ray imaging detector, is typically fixed for any given system, therefore defining the usable imaging field‐of‐view (FOV).

To overcome these limitations, and to help facilitate the scaling up of XPCI methods, we propose multi‐scale tomography with an offset center‐of‐rotation (COR), which enables doubling of the horizontal FOV, without sacrificing pixel size, spatial‐resolution, or X‐ray flux density per detector element. While such scans are well documented with parallel synchrotron beams,^15^, ^16^, ^17^ adaption to cone‐beam systems requires consideration of their divergent imaging geometry. In contrast to a number of laboratory‐based works which utilize offset detectors,^18^, ^19^, ^20^ we instead choose to offset the COR.^21^, ^22^ This maximizes flux efficiency by utilizing the full available X‐ray beam, and in the case of fixed‐divergence sources, enables extended FOV imaging without increasing the system footprint. We demonstrate this method for beam‐tracking X‐ray micro‐CT^12^ of human lung specimens, proving compatibility with systems utilizing optical elements. As a further demonstration of the method's versatility, we also apply the same method to image a punch from the same sample using an X‐ray microscope with free‐space propagation (FSP) XPCI. This demonstrates that the method can be easily adapted to a wide range of existing XPCI and conventional systems.

## 2 MATERIALS AND METHODS

### 2.1 Samples

A sample of resected human lung tissue of approximately 4×4×2 ${\mathrm{cm}}^{3}$ was obtained as surgical surplus with informed patient consent from a left upper lobectomy at University College London Hospital, in a patient diagnosed with squamous‐cell carcinoma of the lung. Approval of all ethical and experimental procedures and protocols was granted by South Central Hampshire B Research Ethics Committee (REC number: 18/SC/0514). The sample was initially fixed in 70% ethanol, followed by dehydration in a series of ascending ethanol concentrations (70%, 80%, 90%, and, 100%) for at least 24 h per stage. The sample was then placed in a bath of hexamethyldisilazane for 4 h, to minimize structural changes that would occur during the final stage of overnight air drying under a fume hood.^23^, ^24^ For imaging with beam‐tracking, the sample was placed inside of a sealed polypropylene container. For the high‐resolution FSP experiment, a 2.7 mm diameter segment of the same sample was removed, and mounted on top of a skewer.

### 2.2 Multi‐scale phase‐contrast microtomography

Experiments were carried out using the NXCT (National research facility for lab‐based X‐ray Computed Tomography) multi‐contrast X‐ray micro‐CT system^25^ (further described in Supplementary Material 1, ^26^). Two exit windows allow simultaneous imaging on two different end‐stations, and an interchangeable anode allows optimization of the system for different samples.

**FIGURE 1:**
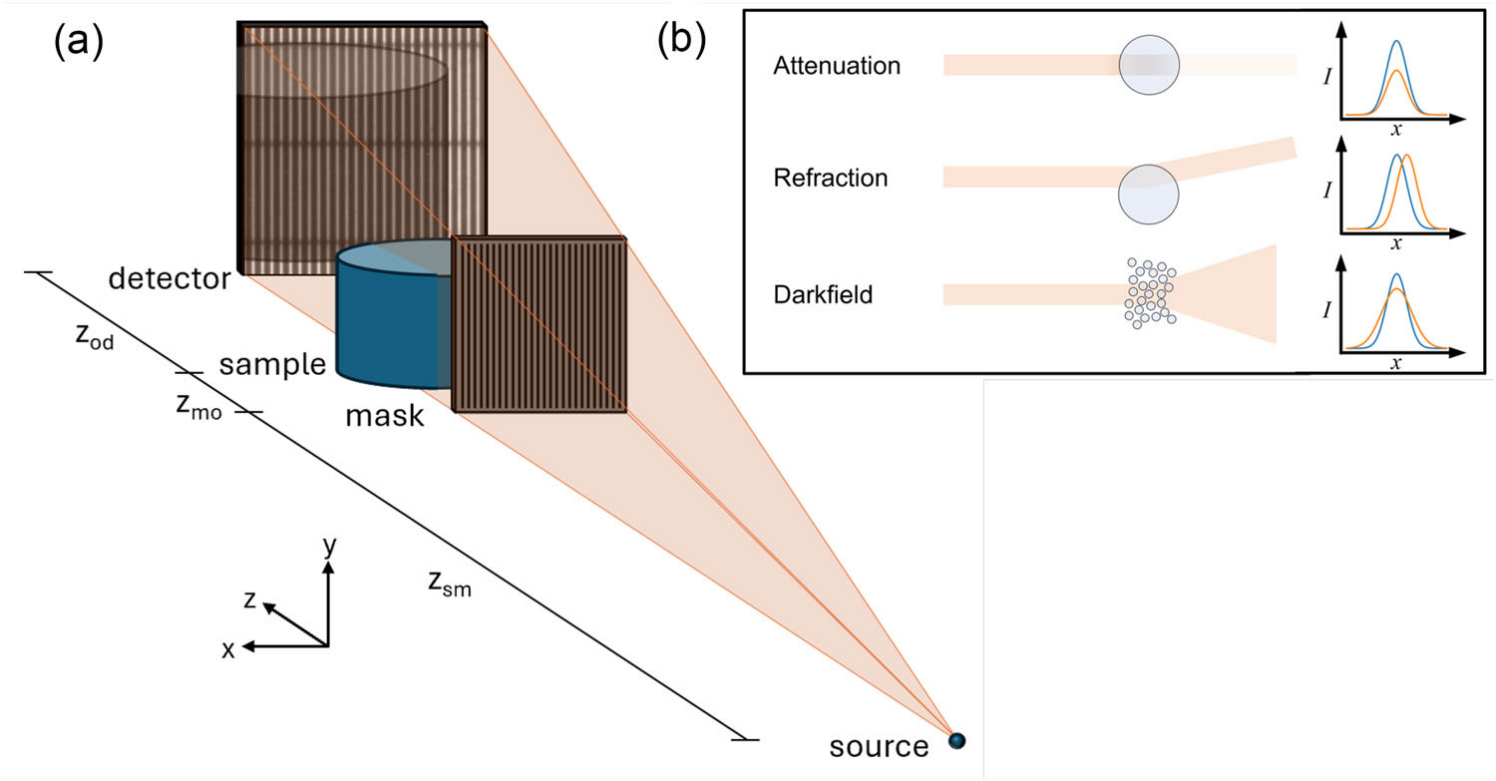
(a) A diagram of the experimental system for beam‐tracking X‐ray microtomography with an offset geometry. The divergent X‐ray beam is emitted from the source and is incident on an absorbing mask which structures the beam into an array of 1D beamlets. The beamlets interact with the sample and after some propagation distance $z_{\text{od}}$ are incident on a high‐resolution detector capable of measuring the perturbations. The diagram is not to scale. (b) The effects of different sample interactions for a single beamlet, with the corresponding 1D detector line profile. Each type of interaction may occur simultaneously, allowing the retrieval of three different contrast channels from a single image.

The full sample was imaged using beam‐tracking, a differential XPCI technique that allows the simultaneous extraction of attenuation, refraction, and dark‐field signals in a single shot.^12^ An absorbing mask consisting of periodic apertures is used to structure the beam into a series of 1D (or 2D^27^, ^28^, ^29^) beamlets, as illustrated in Figure 1(a). The principle of the beam‐tracking technique is that sample‐induced perturbations may be tracked by directly resolving the shape of the beamlets using a high‐resolution detector. By comparing images acquired with and without the sample in the beam (see Supplementary Material 2 ^26^), these small perturbations can be quantitatively retrieved and converted into multi‐contrast images.

The beam‐tracking experiment was carried out using the large FOV end‐station, with a molybdenum anode. By offsetting the COR, the sample plane FOV was doubled by a factor of 1.7× from 2.5 cm to 4.3 cm, sufficient to cover the extent of the sample. Projections were acquired at 2701 angles equally spaced over 360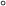, with an 8‐stepped dithered acquisition, resulting in an effective pixel size of 10.5 μm in the x‐direction. An exposure time of 3 s per projection resulted in a total exposure time of 18 h. Attenuation, refraction, and dark‐field contrasts were retrieved from each projection, followed by interlaced stitching. The sample‐free space on the non‐truncated side of the refraction projections provides a Dirichlet boundary condition for integration, thus allowing the exact retrieval of the projected phase. This, alongside the attenuation and dark‐field^30^, ^31^ channels, produces a line integral compatible with quantitative tomographic reconstruction.

The high‐resolution experiment utilized phase‐contrast imaging based on FSP, an established technique in which simple propagation of a sufficiently coherent X‐ray beam converts phase‐shifts into intensity changes.^32^ Under the assumption of a homogenous object, an approximate phase distribution compatible with tomography can be retrieved from the mixed images using an inversion of the transport‐of‐intensity equation.^33^ While being a widely popular technique at synchrotron radiation facilities, FSP based imaging can also be applied to laboratory‐based systems, employing either high‐resolution detectors^34^ or small X‐ray focal spots.^35^

FSP‐based XPCI was carried out using the high‐resolution end‐station of the same system, with a copper anode. An 8 keV monochromatic beam centered on the Kα emission lines was selected using a multilayer flat mirror.^25^ A custom scintillator‐based detector with an effective pixel size of 450 nm enabled high‐resolution imaging and resolving of intensity fringes due to the propagation of the partially coherent beam. Offsetting the COR extended the FOV by a factor of 1.85× from 1.44 mm to 2.70 mm, while maintaining the same pixel size. Projections were acquired at 3001 angles equally spaced over 360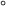. An exposure time of 5 s per projection resulted in a total scan time of just over 4 h. Projections were processed using Paganin single‐distance phase retrieval^33^ with $\frac{\delta }{\beta }$ = 25. The $\frac{\delta }{\beta }$ value was chosen heuristically (favoring under‐retrieval for resolution), using values for soft tissue as an upper bound starting point.

### 2.3 Offset geometry and redundancy weighting

**FIGURE 2:**
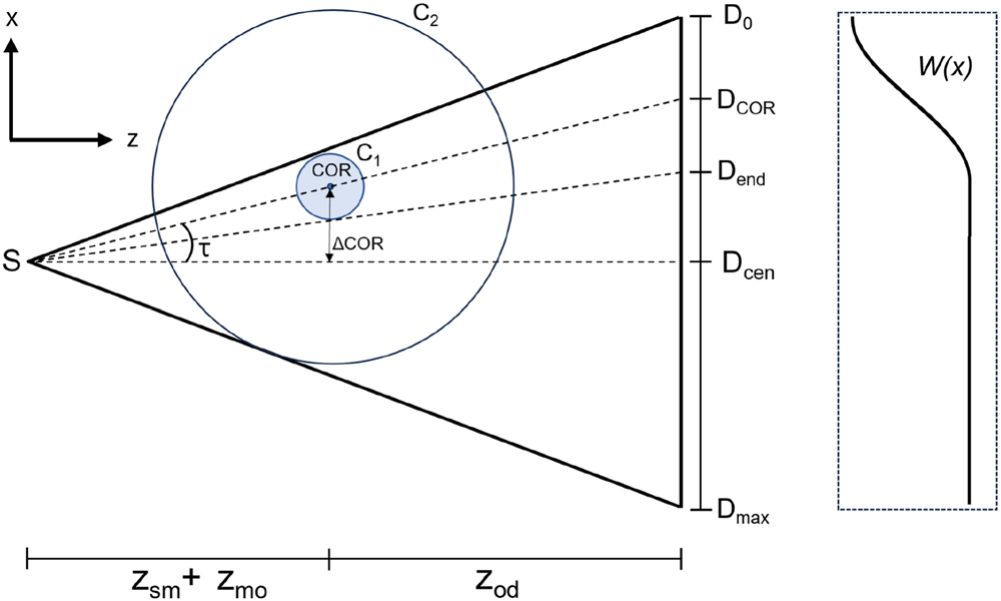
A diagram demonstrating the definitions of the offset tomography geometry. The center‐of‐rotation (COR) is offset by ΔCOR from the axis between the source and detector, creating an angle τ with the detector center. Only the region enclosed by the circle $C_{1}$ remains with the field‐of‐view throughout a 360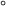 rotation. The remaining region enclosed by $C_{2}$ is seen for some period $<360^{\circ }$ and thus must be weighted differently during the reconstruction. *W(x)* illustrates the weighting function for the demonstrated geometry.

For both scans, the reconstructed FOV was extended by offsetting the sample by a distance ΔCOR along the x‐axis, enabling larger areas to be imaged without sacrificing spatial resolution. By collecting projections as the sample rotates through 360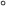, the entirety of the sample within $C_{2}$ in Figure 2 is exposed to the beam. The region covered by $C_{1}$ however is exposed to the beam for the full rotation. A redundancy weighting must therefore be applied to the acquired projections, along x, to correct for the increased density of rays counted within $C_{1}$ compared to $C_{2}$. We adopt a smooth weighting function previously used for complementary offset‐COR short scans,^36^ but instead we apply this principle to a full 360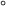 rotation. This requires the acquisition of less data, less mechanical movements of the system, and allows a simpler reconstruction. We describe this as a function of the detector position x a s

(1) $$\begin{array}{ccc} & & W(x)=\\ & & \left\{\begin{array}{cc} 0, & \text{if}\;x\leq D_{0}\\ \text{sgn}\left\{\tau \right\}\sin\left(\frac{\pi }{2}\frac{\tan^{-1}\left(x/R\right)+\tau }{\tan^{-1}\left(pN_{\text{col}}/\left(2R\right)\right)-\left|\tau \right|}\right)+1, & \text{if}\;D_{0}<x\leq D_{\text{end}}\\ 2, & \text{if}\;x\geq D_{\text{end}} \end{array}\right., \end{array}$$

where $N_{\text{col}}$ is the number of detector columns, p is the detector pixel size, and $R=z_{\text{sm}}+z_{\text{mo}}+z_{\text{od}}$. The size of the redundant region is given by $|D_{0}D_{\text{end}}|=|pN_{\text{col}}/2+R\tan\{\tan^{-1}\left[\left(pN_{\text{col}}\right)/\left(2R\right)\right]-2|\tau |\}|$. An example weighting function is plotted in‐line with the corresponding detector positions in Figure 2.

To reduce truncation artefacts^18^, ^20^ without computationally expensive iterative methods,^20^ we apply the redundancy weighting after first applying the ramp filter $\hat{f_{R}}\left(\omega \right)$ to the projections.^37^ Similar to the sinogram extension method,^37^ artefacts due to discontinuities are avoided by padding images with a copy of the width reflected sinogram on the truncated side, and edge values on the non‐truncated side, prior to Fourier transformation. The padded values are then removed before reconstruction. The reconstruction procedure is described by

(2) $$V\left(x,y,z\right)=\text{BP}\left(W\left(x\right)\times F^{-1}\left\{\hat{f_{R}}\left(\omega \right)\times F\left[P(x,y;\alpha )\right]\right\}\right)$$

where BP denotes the backprojection operation that reconstructs the volume V(x,y,z) from projections P(x,y;α), and F and $F^{-1}$ are the forward and inverse Fourier transform operations respectively. The backprojection step is implemented using a vector backprojection algorithm,^38^ allowing the exact custom geometry to be defined. Easily adaptable code to calculate and apply offset‐geometry weights, alongside a demonstration on simulated data, is made publicly available.^39^

## 3 RESULTS AND DISCUSSION

**FIGURE 3:**
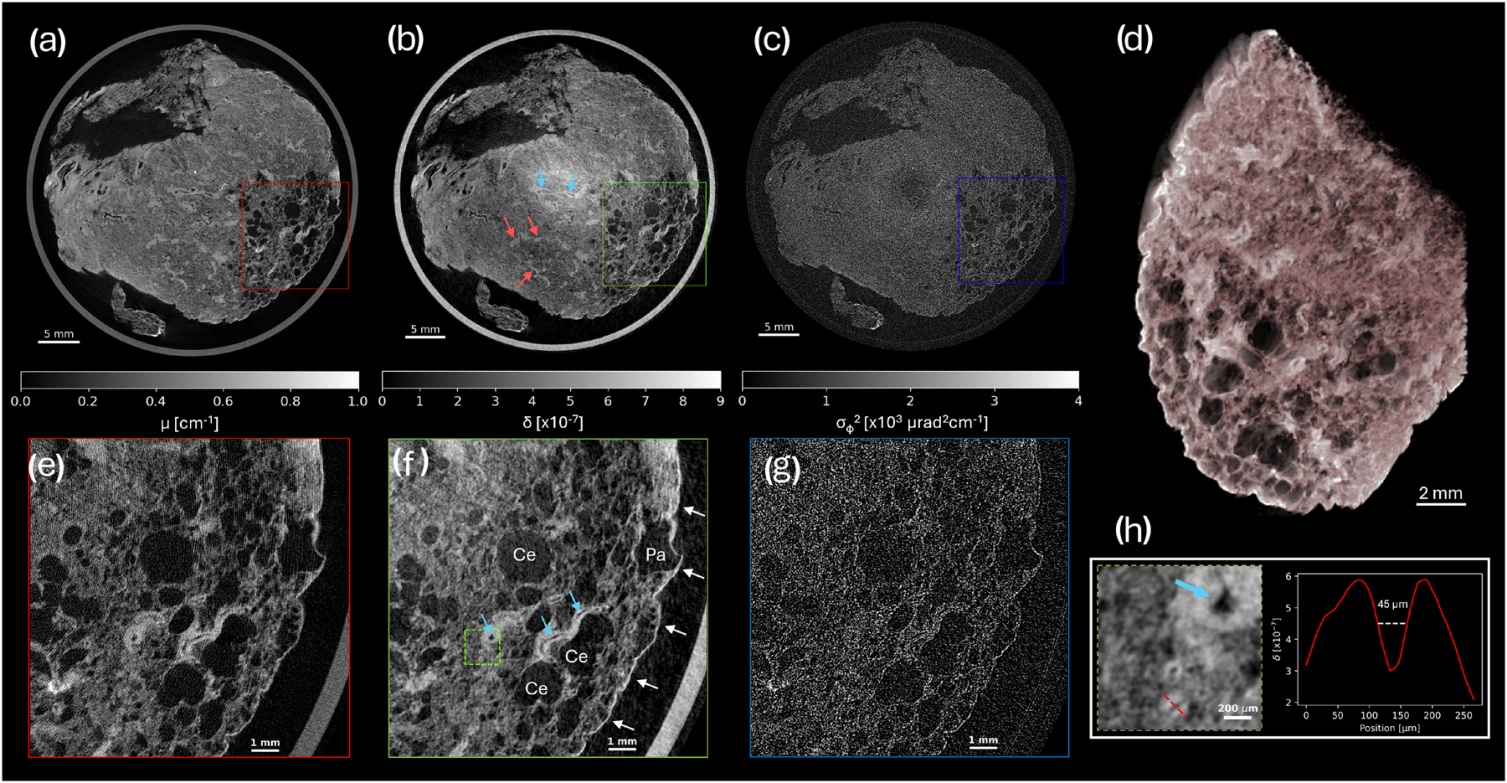
Axial slices of the offset beam‐tracking tomography in attenuation (a), phase (b), and dark‐field (c) contrast channels. Volumetric information allows the network of arteries (blue arrows) and veins (red arrows) to be traced through the tissue. Zoomed views at the positions indicated by the coloured boxes also display the attenuation (e), phase (f), and dark‐field (g) channels, where it is evident that the phase channel enables the visualisation of fine, low‐contrast features. The phase zoom (f) illustrates the well defined pleura (white arrows) and examples of fine pulmonary arteries (blue arrows), as well as markers of disease: enlarged air spaces indicative of centrilobular (Ce) and paraseptal (Pa) emphysema. A further zoom (h), taken from the position indicated by the dashed green box (f), demonstrates the ability to resolve fine features. A line profile is taken through an arterial lumen of several tens of micrometres, which can be traced back to the larger vessel indicated by the red arrows in (b). The region in (f) is rendered as a volume (d), where many large emphysematous air spaces are made visible.

Figure 3 illustrates the reconstruction of the beam‐tracking scan using the proposed method. Axial slices of the attenuation (3a), phase (3b), and dark‐field (3c) contrast channels are shown, alongside corresponding zooms (3e,f,g). Volumetric information allows the tracing of pulmonary vasculature, with both venous (red arrows) and arterial (blue arrows) systems labelled in Figure 3(b). Fine, low‐contrast details are made more easily visible in the phase channel, with a number of these details annotated on Figure 3(f). In particular, a network of fine vasculature can be seen, which can be followed back through the volume to the vessels labelled in Figure 3(b). A further zoom and line profile (3h) demonstrates an arterial lumen with a full‐width‐at‐half‐maximum (consisting of the convolution of the lumen profile with the system spatial resolution) of 45 μm. Note that for this sample, much of the venous system appears with a clear air‐filled lumen, likely as a consequence of the sample preparation protocol. A number of enlarged air spaces indicative of centrilobular (Ce) and paraseptal (Pa) emphysema are visible in the slices, and particularly also in the volume rendering (3d). These lesions are both markers of disease that are consistent with the patient's smoking history.

**FIGURE 4:**
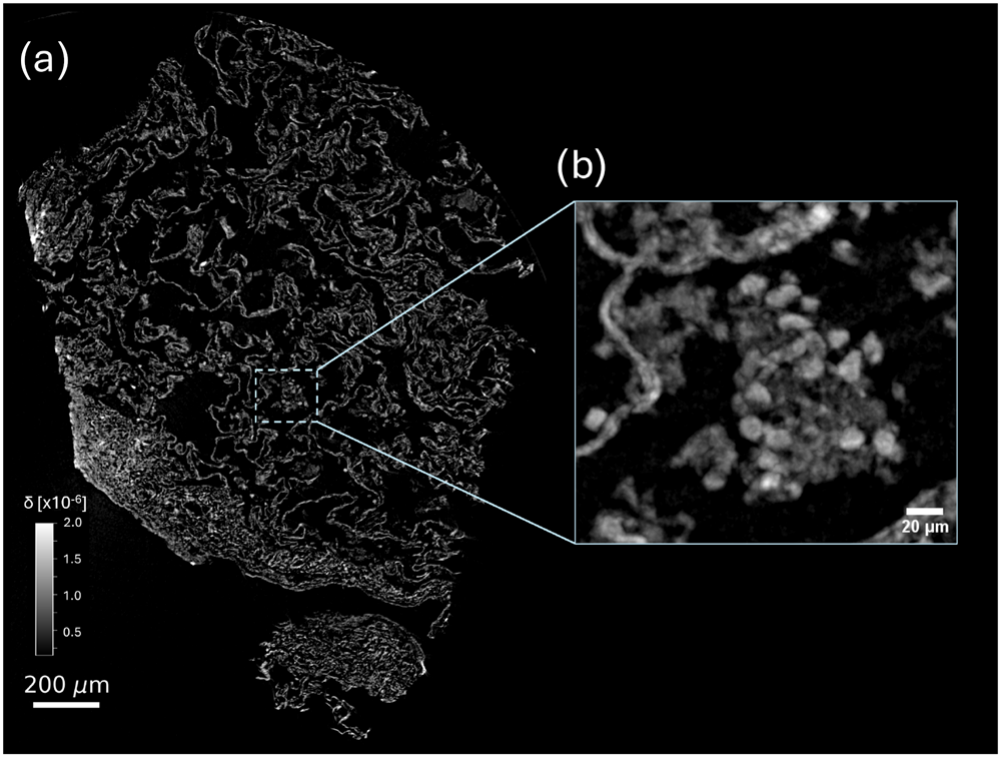
Axial slice (a) of the high‐resolution tissue section, illustrating with greater detail the mesh of collapsed alveoli. Dispersed through the tissue are clusters of white blood cells (b), highlighted by a 9 μm thickness maximum intensity projection.

Figure 4 illustrates the reconstruction of the high‐resolution scan using the proposed method. The axial slice in Figure 4(a) shows how, at higher resolution, the collapsed and closely spaced alveolar septa visible in Figure 3 are now better defined. A range of notable histological structures are discernible at this resolution. In particular, clusters of white blood cells (possibly macrophages) are interspersed throughout the tissue. Panel 4b shows a maximum intensity projection equivalent to 9 μm thickness (comparable with histology slide thicknesses), in which a number of these cells are visible, clustered around a clump of amorphous debris.

We have demonstrated a number of features that may only be observed by acquiring scans at different length‐scales. Some of the enlarged air spaces in Figure 3 are larger than the entire FOV of the high‐resolution system (even in combination with offset scanning), and thus could not possibly measured. Similarly, measurement of clusters of white blood cells is only possible using the high‐resolution setup. The possibility to investigate different length scales using the same instrument could allow selective zooming of areas chosen based upon the large FOV overview. In all cases, the achievable FOV can be extended using the offset scanning method. It is particularly valuable that the analytical weighted reconstruction allows the retrieval of accurate volumes, without the necessity to use potentially slow iterative methods. This factor is of particular importance if the technique is applied in fields where rapid reconstructions are required, such as interoperative imaging, for which XPCI has already shown positive outcomes.^40^

Despite these advantages, several limitations should be noted. The offset‐geometry results in a non‐uniform sample coverage and thus dose‐distribution. While dose itself is potentially a larger concern for in‐vivo studies, this would also result in an uneven CNR across the FOV. Similarly, angular sampling varies across the object. Both factors can reduce the visibility of features further away from the COR. As the extension ratio is increased, the number of required projections also increases, in line with the Nyquist criterium. Thus for full sampling, increased FOV comes at the cost of longer scan times.

We also note the presence of low frequency artefacts in the center of the phase and dark‐field reconstructions. These are caused by signal bias in the retrieval process, which arises due to the spatially varying detector point spread function. Similar effects have been reported previously, along with methods for correction.^41^

While the multi‐contrast capabilities were demonstrated using a beam‐tracking approach, the same principles can be extended to a range of multi‐contrast XPCI systems such as grating‐interferometry,^10^ edge‐illumination,^11^ and speckle‐tracking.^42^ In particular, it should be noted that offset scanning does not require any specialized adaptions other than the ability to translate the rotation stage, and thus is inherently compatible and stackable with FOV‐extension methods such as tiled‐gratings^43^, ^44^ and scanning‐based methods.^45^, ^46^ The methods shown have been developed in the context of lung specimen imaging, but are widely applicable across XPCI and conventional CT of a range of samples, including soft‐tissues and light materials. The large degree of generalization could open application to other methods in which a cone‐beam reconstruction is required, including advanced multi‐modal imaging,^47^ and even holotomography using focused synchrotron radiation.^3^

## 4 CONCLUSION

We have demonstrated a versatile methodology for multi‐scale and multi‐contrast X‐ray microtomography. By utilizing an offset COR, it was shown that the achievable FOV at each scale can be doubled, enabling scans of larger samples at a given resolution. Applied to human lung tissue, the multi‐scale approach enabled the visualization of a range of features from the macro to cellular scale. The methods demonstrated here are easily adapted to conventional and phase‐contrast systems, and will enable an increase in the range and size of samples that can be investigated using X‐ray tomography.

## CONFLICT OF INTEREST STATEMENT

The authors declare no conflicts of interest.

## Supporting information

Supporting Information

## Data Availability

The data that support the findings of this study are available from the corresponding author upon reasonable request.
