# Supplementary material for: Multi‐contrast X‐ray microtomography of human lung specimens with an extended field‐of‐view
Source: Med Phys. 2026 Feb 19;53(2):e70335. doi: 10.1002/mp.70335 (PMC12919702; doi:10.1002/mp.70335)
Supplement: Supplementary file 1 — Supporting Information [file MP-53-0-s001.pdf]

Supplementary material: Multi-contrast X-ray microtomography of human lung specimens with an extended field-of-view

Harry Allan[1,2,*], Adam Doherty[1,2], Carlos Navarrete-León[1,2], Oriol Roche i Morgó[1,2,7], Yunpeng Jia[1,2,8], Charlotte Percival[3], Zoe Hagel[3], Kate E J Otter[3], Chuen Ryan Khaw[3], Kate HC Gowers[3], Helen Hall[3,9], Sam M Janes[3], Fleur Monk[4], David Moore[4,5], Ryoko Egashira[6], Joseph Jacob[3,6], Marco Endrizzi[1,2]

[1]*Department of Medical Physics and Biomedical Engineering, University College London, London, UK, WC1E 6BT*

[2]*X-ray Microscopy and Tomography Laboratory, The Francis Crick Institute, London, UK, NW1 1AT*

[3]*UCL Respiratory, University College London, London, UK, WC1E 6JF*

[4]*Department of Cellular Pathology, University College London Hospitals NHS Foundation Trust, London, UK, W1T 4EU*

[5]*CRUK Lung Cancer Centre of Excellence, UCL Cancer Institute, University College London, London, UK, WC1E 6DD*

[6]*Hawkes Institute, University College London, London, UK, WC1V 6LJ*

1 NXCT multi-contrast X-ray micro-CT system

Experiments were carried out using the NXCT (National research facility for lab-based X-ray Computed Tomography) multi-contrast X-ray micro-CT system [1]. The system features a Rigaku MicroMax 007-HF rotating anode X-ray source, with a focal spot size of 70 µm. Two exit windows allow simultaneous imaging on two different end-stations, both seeing the same effective focal spot. An interchangeable anode, with options of Mo and Cu, allows optimisation of the system for different samples.

1.1 Beam-tracking microtomography

For the beam-tracking experiment, the source was operated with a molybdenum anode with 50 kV tube voltage and 24 mA current. The beam was filtered with a total of 0.18 mm of aluminium, to increase penetration through the sample. A custom-built lens-coupled scintillator detector, with an effective pixel size of 15 µm and a FOV of $\sim$ 30 mm, was integrated into the system. The X-ray beam was structured using a 1D absorption mask with apertures of 10 µm, and a period of 79 µm. The system geometry was set to $z_{sm} = 820$ mm, $z_{mo} = 50$ mm, and $z_{od} = 170$ mm. This resulted in a native planar FOV at the sample plane of 2.5 cm. Multi-contrast images with horizontal resolution given by the mask period can be acquired in a single shot. Alternatively, dithering the position of the mask relative to the sample enables aperture driven resolution [2].

1.2 High-resolution free-space propagation microtomography

While the beam-tracking technique has also been applied at the microscale [3], the reduced system efficiencies at these scales makes tomography very time consuming, and puts further constraints on the positional stability of the system. For this reason, and to further demonstrate the versatility of the technique across a range of methods, FSP-based XPCI was carried out using the high-resolution end-station. The source was operated at 40 kV and 30 mA, with a copper anode. An 8 keV monochromatic beam centred on the Kα lines was selected using a multilayer flat mirror [1]. A different custom-built lens-coupled scintillator detector, with an effective pixel size of 450 nm and a horizontal FOV of 1.44 mm, was integrated into the system. The detector was placed 390 mm away from the source, with the propagation distance set to $z_{od} = 8$ mm. By using high-resolution detectors, phase-contrast imaging is possible even with relatively large focal spots, such as the 70 µm used in this work. This concept has been applied elsewhere with sources as large as 350 µm [4].

---

[7]Now with the Diamond Light Source, Harwell Science and Innovation Campus, Didcot, UK, OX11 0DE.
[8]Now with the Department of Life Sciences, Birmingham City University, Birmingham, UK, B4 7BD.
[9]Now with the Department of Respiratory Medicine, King's College Hospital, London, UK, SE5 9RS.

2 Multi-contrast retrieval in X-ray beam tracking imaging

The image retrieval process involves the extraction of horizontal 1D line profiles from the recorded projections, each corresponding to individual beamlets at indexed image positions (x, y). Sample-induced attenuation causes a decrease in the intensity of the beamlet, refraction causes an angular deflection of the beamlet, and dark-field causes a broadening of the beamlet. These are reflected as changes in the measured areas $A(x, y)$, centroids $c(x, y)$, and widths $\sigma(x, y)$ respectively of the extracted beamlet profiles upon the detector. Beamlet areas and widths are calculated numerically as the zeroth and second central moments of the measured beamlet intensity distributions. The shift in the centroid Δc is estimated as the location of the maximum of the cross-correlation of the min-max normalised sample $I_S(x_i)$ and flat $I_F(x_i)$ beamlet intensity distributions

$$\Delta c = \underset{\epsilon}{\text{argmax}} \left(\sum_i I_S(x_i) I_F(x_i + \epsilon) \right), \quad (1)$$

where ϵ is the shift parameter, and $_i$ is the index along the 1D profile. The cross correlation is estimated first for integer $-w < \epsilon < w$, where w is the beamlet half-width. This is followed by sub-pixel localisation by analytical parabolic interpolation of the maximisation landscape [5].

Comparison of the flat and sample images allows extraction of the sample transmission, refraction angle, and dark-field signals by

$$T(x, y) = \frac{A_S(x, y)}{A_F(x, y)}, \quad (2)$$

$$\theta_R(x, y) = \frac{\Delta c(x, y)}{\text{z}_{\text{od}}}, \quad (3)$$

$$\sigma^2(x, y) = \frac{\sigma_S^2(x, y) - \sigma_F^2(x, y)}{\text{z}_{\text{od}}^2}, \quad (4)$$

where z_{od} is the propagation distance between the sample and detector, and the subscripts $_S$ and $_F$ refer to quantities calculated using images with and without the sample respectively. Correction of sample images by the corresponding flat images corrects for any illumination differences such as heel effects.

The retrieved transmission $T(x, y)$ is related to the line integral through the object $\int_o dz$ of the 3D distribution of the linear attenuation coefficient μ_0 by

$$-\log(T(x, y)) = \int_o \mu_0(x, y, z)\, dz. \quad (5)$$

The retrieved refraction angle is related to the derivative of the projected phase shift by $\theta_R(x, y) = \frac{1}{k} \frac{\partial \Phi(x,y)}{\partial x}$, where $\Phi(x, y)$ is the phase shift and k is the wavenumber. Retrieving the projected phase through unidirectional integration allows the construction of the line integral

$$-\frac{\Phi(x, y)}{k} = \int_o \delta(x, y, z)\, dz. \quad (6)$$

It has been shown previously that beam-tracking and edge-illumination retrieve a quantitative dark-field signal that is linear with thickness [6, 7], and thus we may finally also construct the line integral

$$\sigma^2 = \int_o \sigma_\phi^2(x, y, z)\, dz, \quad (7)$$

where σ_ϕ^2 is the linear scattering coefficient. It follows that each of the retrieved quantities is consistent with tomographic reconstruction, allowing the recovery of their 3D volumetric distributions.

When fast scans are required, multi-contrast volumes may be reconstructed from a single image per projection angle, delivering spatial resolution limited by the mask period, yet still remaining quantitative [8]. When higher spatial resolution is demanded, dithering may be employed, in which multiple images are acquired while the mask is translated in sub-period steps. The individual retrieved images are then stitched together in an interlaced pattern, leading to approximately aperture-driven resolution [2] perpendicular to the mask apertures. The resolution parallel to the mask apertures is dependent on the quadrature sum of the detector resolution and the magnified source size, as in regular X-ray projection imaging. To account for the unequal pixel size, the images are interpolated onto a square grid (to a pixel size of the smallest of either the mask step or the magnified vertical detector pixel) using bicubic interpolation, prior to reconstruction.

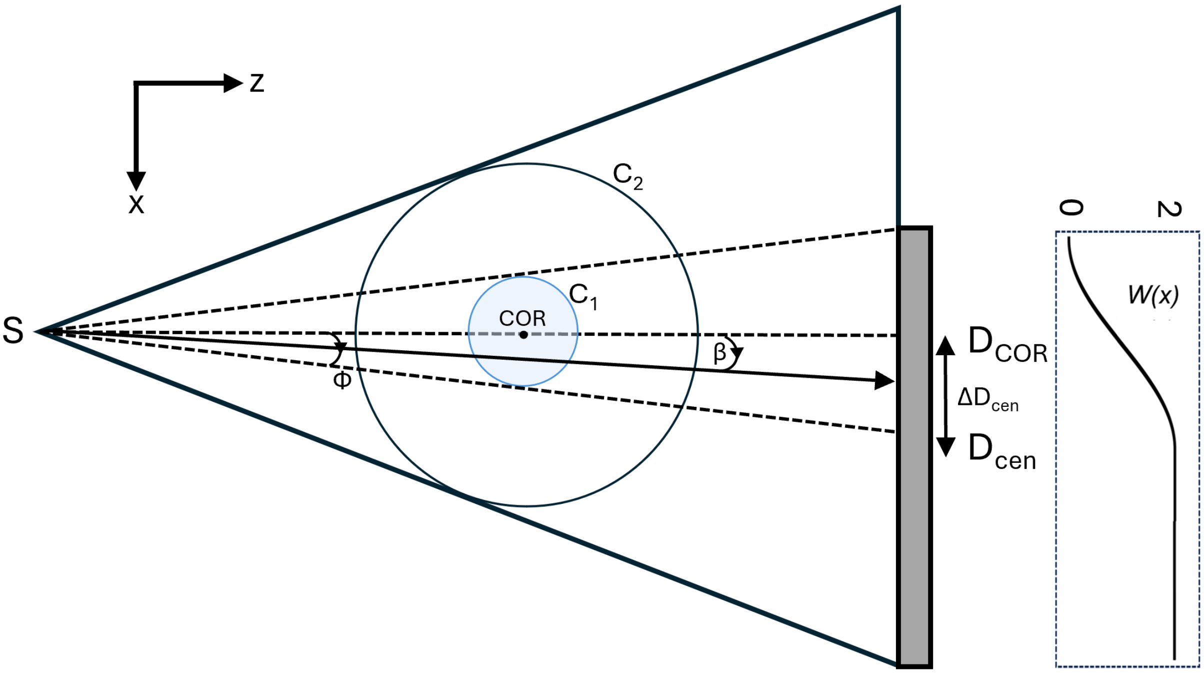

Figure 1: Offset detector geometry, in which the line **S COR** is perpendicular to the offset detector. The region of the sample within the circle C_1 remains within the field-of-view for the full rotation, while the region enclosed within C_2, but excluding C_1, remains in the field-of-view for $< 360°$.

3 Derivation of redundancy weights

Consider a cone-beam beam geometry with its origin at the centre-of-rotation COR, as illustrated in Figure 2. In the sample reference frame, the circular tomographic trajectory with projection angles α is described by the relative positions of the source and (centre of) detector, indicated by $\mathbf{S}(x, y, z)$ and $\mathbf{D_{cen}}(x, y, z)$, respectively. For brevity, the coordinate dependence (x, y, z) is henceforth omitted. To define these positions, by the conventions of the Astra Toolbox [9], we use the opposing unit vectors $\mathbf{V_s} = [\sin(\alpha), 0, -\cos(\alpha)]$, and $\mathbf{V_d} = [-\sin(\alpha), 0, \cos(\alpha)]$, where α is the angle of a given projection. Let us begin with the offset detector geometry illustrated in Figure 1, where a given ray makes an angle β with the the line **S**COR.

The region of the sample enclosed by the circle C_1 remains inside of the FOV for the full 360° rotation. Meanwhile, the region enclosed within C_2, excluding C_1, remains in the field-of-view for $< 360°$. The rays $-\Phi \leq \beta \leq \Phi$ (where Φ marks the edge of the redundant region) have complementary rays at opposite α, and must be weighted for their redundancy. For the offset detector geometry, the redundancy weighting function $W(x)$ must fulfill $W(D_{COR} + n)$ + $W(D_{COR} - n)$ = 2 [10], where the points $D_{COR} + n$ and $D_{COR} - n$ are detector positions symmetric about the projected COR D_{COR}.

Furthermore, the weighting function in the redundancy region should be smooth and absent of discontinuities (thus the function and its derivatives should be smooth) [11]. While this may be satisfied by a number of weighting functions, one such example for the offset detector case is the sinusoidal weighting function [12]

$$W(\beta) = \sin\left(\frac{\pi}{2}\frac{\beta}{\Phi}\right) + 1, \qquad -\Phi \leq \beta \leq \Phi. \tag{8}$$

Let us now consider the offset COR geometry in Figure 2. Here, the line $\mathbf{SD_{cen}}$ is parallel to the optical axis, and instead, the COR is offset. We can write the respective source and detector positions in this geometry as

$$\mathbf{S} = (z_1)\mathbf{V_s} + (\Delta\text{COR})\mathbf{U}, \tag{9}$$

$$\mathbf{D_{cen}} = (z_{od})\mathbf{V_d} + (\Delta\text{COR})\mathbf{U}, \tag{10}$$

where ΔCOR is the offset (with sign following the geometry conventions in Figure 2) of the COR along x, and $\mathbf{U} = [\cos(\alpha), 0, \sin(\alpha)]$ is the unit vector describing the detector pixel orientation, perpendicular to the line $\mathbf{SD_{cen}}$.

For the offset COR geometry, the redundancy weighting must be symmetric in angle β about D_{COR}, giving $W(D_{COR} + \beta)$ + $W(D_{COR} - \beta)$ = 2 [10]. Let us introduce $\gamma = \tan^{-1}(x/R)$ as the angle that a ray makes with the

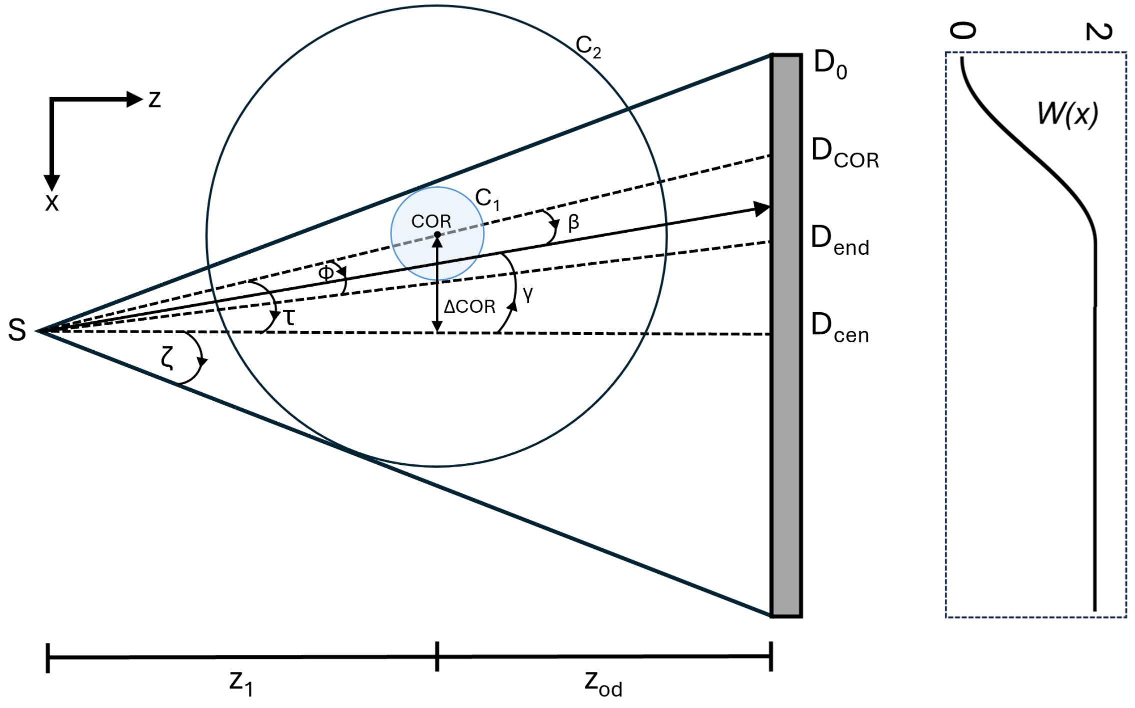

Figure 2: A diagram demonstrating the definitions of the offset COR geometry. The centre-of-rotation (COR) is offset by ΔCOR from the axis between the source and detector, creating an angle τ with the detector centre. Only the region enclosed by the circle C_1 remains with the field-of-view throughout a 360° rotation. The remaining region enclosed by C_2 is seen for some period <360° and thus must be weighted differently during the reconstruction. W(x) illustrates the weighting function for the demonstrated geometry.

optical axis. Transformation of γ, from the optical axis centred coordinate system (where γ = 0° is parallel to $\mathbf{SD}_{\text{cen}}$) to the **S**COR centred coordinate system (where β = 0° is parallel to **S**COR), allows offset COR weights to be derived from offset detector weights. Following [13], the offset COR geometry can be considered as a rigid rotation of the source and detector system by an angle $\tau = -\tan^{-1}(\Delta\text{COR}/z_1)$ about **S**, thus giving the transformation $\beta = \gamma + \tau$. The angular range Φ of the redundancy region is similarly transformed, and is given by $\Phi = \zeta - |\tau|$, with $\zeta = \tan^{-1}(pN_{\text{col}}/(2R))$ as the half-angle of the measured X-ray cone, where p is the detector pixel size, N_{col} is the number of detector columns, and $R = z_1 + z_{\text{od}}$.

From Equation 8, make the substitution $\beta = \gamma + \tau$, and $\Phi = \zeta - |\tau|$, resulting in

$$W(\gamma) = \begin{cases} 0 & \text{, if } \gamma \leq -\zeta \\ \sin\left(\frac{\pi}{2}\frac{\gamma+\tau}{\zeta-|\tau|}\right) + 1 & \text{, if } -\zeta < \gamma \leq \Phi - \tau \ . \\ 2 & \text{, if } \gamma \geq \Phi - \tau \end{cases} \tag{11}$$

Making further substitutions of τ and ζ, alongside adding the multiplier sng$\{\tau\}$ to make the equation offset direction agnostic, we state the weighting function in terms of the detector position x as

$$W(x) = \begin{cases} 0 & \text{, if } x \leq D_0 \\ \text{sng}\{\tau\}\sin\left(\frac{\pi}{2}\frac{\tan^{-1}(x/R)+\tau}{\tan^{-1}(pN_{\text{col}}/(2R))-|\tau|}\right) + 1 & \text{, if } D_0 < x \leq D_{\text{end}} \ , \\ 2 & \text{, if } x \geq D_{\text{end}} \end{cases} \tag{12}$$

The sinusoidal weighting is thus applied in the range $D_0 < x \leq D_{\text{end}}$, where the size of this redundant region is given by $|D_0 D_{\text{end}}| = |pN_{\text{col}}/2 + R\tan\{\tan^{-1}[(pN_{\text{col}})/(2R)] - 2|\tau|\}|$. An example weighting function is plotted inline with the corresponding detector positions in Figure 2.

3.1 Reconstruction

To reduce truncation artefacts, we apply the redundancy weighting after first applying the ramp filter $\hat{f}_R(\omega)$ to the projections [10]. Similar to the sinogram extension method [10], artefacts due to discontinuities are avoided by padding images with a copy of the width reflected sinogram on the truncated side, and edge values (normally

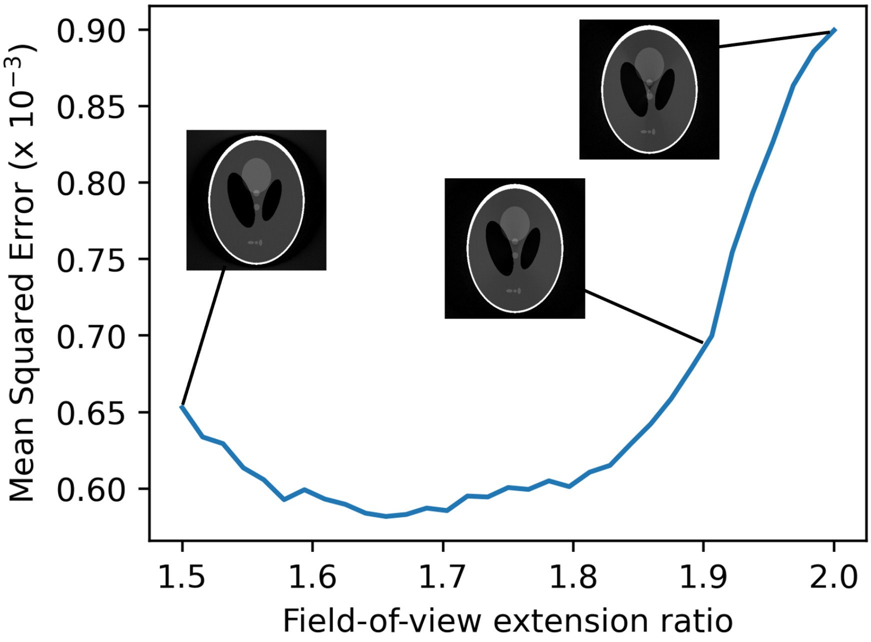

Figure 3: Mean squared error of the reconstructed slices as a function of field-of-view extension ratio. Illustrative slices are shown at ratios of 1.5x, 1.91x, and 2x.

zeros) on the non truncated side, prior to Fourier transformation. The padded values are then removed before reconstruction. The reconstruction procedure is described by

$$V(x,y,z) = \text{BP}\left(W(x) \times \mathcal{F}^{-1}\{\hat{f}_R(\omega) \times \mathcal{F}[P(x,y;\alpha)]\}\right), \tag{13}$$

where BP denotes the backprojection operation that reconstructs the volume $V(x,y,z)$ from projections $P(x,y;\alpha)$, and $\mathcal{F}$ and $\mathcal{F}^{-1}$ are the forward and inverse Fourier transform operations respectively. The back-projection step is implemented using vector backprojection algorithm in the Astra Toolbox [9]. Easily adaptable code to calculate and apply offset-geometry weights, alongside a demonstration on simulated data, is made publicly available [14].

Prior to weighting and reconstruction, sinograms were processed with a stripe removal algorithm to reduce the appearance of ring artefacts. Briefly, stripes were identified by averaging sinograms along the angle direction and subtracting the low-pass filtered component. The resulting profile was then used to normalise the sinograms by subtraction. While broadly successful, some faint residual rings may be visible in the attenuation slice of Figure 3e of the main manuscript.

4 Domain of validity and accuracy under large truncations

4.1 Accuracy under large extension ratios

Previous works [10] have shown that post-convolution weighting of displaced detector scans yields accurate and artefact-free reconstruction up to larger extension ratios (and thus more severe truncation) than the pre-convolution weighting method. To explore how this applies to the proposed offset COR method, the tomography of the central slice of a Shepp-Logan phantom was simulated, using progressively larger extension ratios. Forward projection was simulated using the Astra Toolbox [9], with z_1 = 800 mm, z_{od} = 200 mm, and p = 20 µm. The extension ratio was varied between 1.5x and 2x, with 1401 projections (much larger than Nyquist criterium, to avoid sampling dependence) in each case. The volume was subsequently reconstructed using the proposed reconstruction method.

The mean squared error (MSE) was calculated for each extension ratio, inside of a mask defining the extent of the sample. The results are plotted in Figure 3, with the reconstructed slice at three positions indicated. The visual reconstruction quality remains passable across the range, with progressively increasing MSE at higher extension ratios. Visually, the reconstruction at the maximum extension (2x) begins to show some small streak artefacts, arising from the lack of weighting smoothness (the weighting function $W(x) = 2$ across all x).

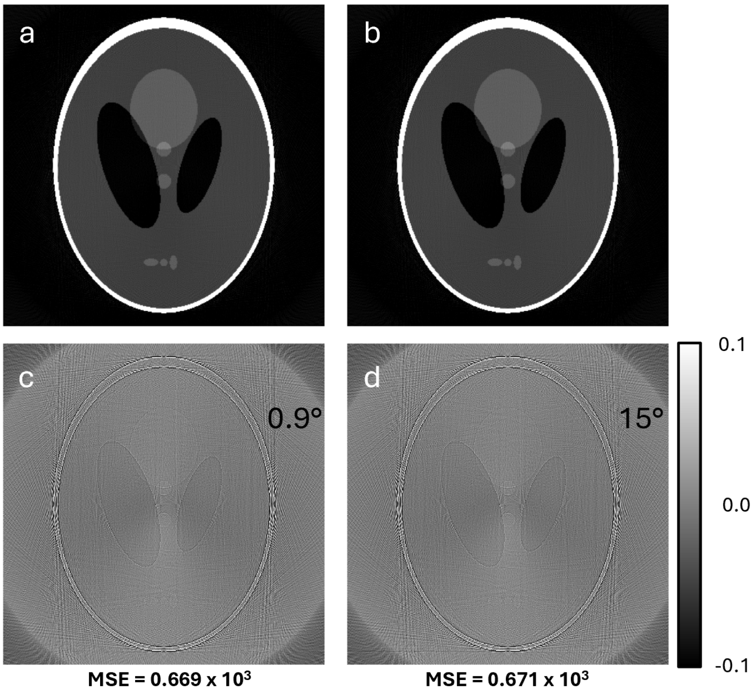

Figure 4: Reconstructed slices from simulations with 0.9° (a) and 15° (b) cone angles. Both reconstructions show comparable accuracy, as illustrated by the similar MSEs and error maps (c and d, respectively).

4.2 Accuracy for large cone angles

The proposed method has been experimentally demonstrated for a system with cone half angle of $\sim$0.9°. Alternatively, micro-CT and XPCI are often applied using microfocus x-ray sources, which commonly come with much larger cone angles. To confirm the validity of the method for both large and small cone angles, the same simulation was repeated at two different cone angles. Geometrical magnification was kept fixed at 1.25, while changing the total system length to vary the cone half angle from 0.9° to 15° (comparable to systems such as [15, 16]). The extension ratio was kept fixed at 1.8x. The reconstructed slices are shown in Figure 4, illustrating the comparable reconstruction accuracy in both cases.

[1] O. Roche i Morgó, Y. Jia, H. Allan, A. Doherty, C. Navarrete-León, A. Astolfo, L. Jiang, J. D. Ferrara, and M. Endrizzi, "A new user facility with flexible multi-scale, multi-contrast micro-ct capabilities," in *Developments in X-Ray Tomography XV*, vol. 13152, p. 1315209, SPIE, 2024.

[2] P. C. Diemoz, F. A. Vittoria, and A. Olivo, "Spatial resolution of edge illumination x-ray phase-contrast imaging," *Optics express*, vol. 22, no. 13, pp. 15514–15529, 2014.

[3] M. Esposito, L. Massimi, I. Buchanan, J. D. Ferrara, M. Endrizzi, and A. Olivo, "A laboratory-based, low-energy, multi-modal x-ray microscope with user-defined resolution," *Applied Physics Letters*, vol. 120, no. 23, 2022.

[4] M. Esposito, N. Schieber, A. Olivo, Y. Schwab, and M. Endrizzi, "Laboratory-based x-ray phase contrast microscopy system for targeting in unstained soft-tissue samples," *Physical Review Research*, vol. 7, no. 1, p. 013037, 2025.

[5] Y. Kashyap, A. Agrawal, M. Shukla, H. Wang, and K. Sahwney, "A comparative study of sub-sampling methods in x-ray speckle interferometry based phase contrast imaging using synchrotron radiation source," *Nuclear Instruments and Methods in Physics Research Section A: Accelerators, Spectrometers, Detectors and Associated Equipment*, vol. 1070, p. 170042, 2025.

[6] M. Endrizzi, F. Vittoria, L. Rigon, D. Dreossi, F. Iacoviello, P. Shearing, and A. Olivo, "X-ray phase-contrast radiography and tomography with a multiaperture analyzer," *Physical review letters*, vol. 118, no. 24, p. 243902, 2017.

[7] A. Doherty, S. Savvidis, C. Navarrete-León, M. F. Gerli, A. Olivo, and M. Endrizzi, "Edge-illumination x-ray dark-field tomography," *Physical Review Applied*, vol. 19, no. 5, p. 054042, 2023.

[8] C. Hagen, P. Diemoz, M. Endrizzi, and A. Olivo, "The effect of the spatial sampling rate on quantitative phase information extracted from planar and tomographic edge illumination x-ray phase contrast images," *Journal of Physics D: Applied Physics*, vol. 47, no. 45, p. 455401, 2014.

[9] W. Van Aarle, W. J. Palenstijn, J. Cant, E. Janssens, F. Bleichrodt, A. Dabravolski, J. De Beenhouwer, K. J. Batenburg, and J. Sijbers, "Fast and flexible x-ray tomography using the astra toolbox," *Optics express*, vol. 24, no. 22, pp. 25129–25147, 2016.

[10] P. S. Cho, A. D. Rudd, and R. H. Johnson, "Cone-beam ct from width-truncated projections," *Computerized medical imaging and graphics*, vol. 20, no. 1, pp. 49–57, 1996.

[11] D. L. Parker, "Optimal short scan convolution reconstruction for fan beam ct," *Medical physics*, vol. 9, no. 2, pp. 254–257, 1982.

[12] G. Wang, "X-ray micro-ct with a displaced detector array," *Medical physics*, vol. 29, no. 7, pp. 1634–1636, 2002.

[13] G. Belotti, S. Rit, and G. Baroni, "Extension of the cone-beam ct field-of-view using two short scans with displaced centers of rotation," in *7th International Conference on Image Formation in X-Ray Computed Tomography*, vol. 12304, pp. 81–86, SPIE, 2022.

[14] H. Allan and M. Endrizzi, "Offset cone-beam ct reconstruction code." `https://github.com/Hallan99/offset-cone-beam-CT-reconstruction`, 2024.

[15] F. A. Vittoria, G. K. Kallon, D. Basta, P. C. Diemoz, I. K. Robinson, A. Olivo, and M. Endrizzi, "Beam tracking approach for single–shot retrieval of absorption, refraction, and dark–field signals with laboratory x–ray sources," *Applied Physics Letters*, vol. 106, no. 22, p. 224102, 2015.

[16] H. Wang, Y. Kashyap, and K. Sawhney, "From synchrotron radiation to lab source: advanced speckle-based x-ray imaging using abrasive paper," *Scientific reports*, vol. 6, no. 1, p. 20476, 2016.
